# Optimization of seat allocation with fixed prices: An application of railway revenue management in China

**DOI:** 10.1371/journal.pone.0231706

**Published:** 2020-04-21

**Authors:** Wuyang Yuan, Lei Nie

**Affiliations:** 1 School of Traffic and Transportation, Beijing Jiaotong University, Beijing, China; 2 State Key Laboratory of Rail Traffic Control and Safety, Beijing Jiaotong University, Beijing, China; Shandong University of Science and Technology, CHINA

## Abstract

China Railway Corporation (CRC) has been paid more attention to passenger transportation revenue, with its increase proportion in transportation revenue. Due to the price regulation, the only way CRC can improve ticket sale profit is to find a best seat allocation scheme. This study focuses on the optimization of railway revenue management problem in China with consideration of i) customer behaviors including their arrival and purchase preferences, ii) a specific ticket booking mechanism called “seat-based control”. To evaluate the performance of seat-based control, we build a Discrete-Time Markov Chain model to describe the ticket reservation process and then design a genetic algorithm to find approximate solutions. The performance of proposed method is tested in two experiments with two other benchmarks. Finally, we apply it to practical data of the Nanning-Guangzhou high-speed railway line.

## 1 Introduction

With the expansion of the high-speed railway (HSR) network in China, the ticket sale income of China Railway Corporation has rapidly increased. Consider the Beijing-Shanghai HSR line, for example, which opened in 2011 and became profitable in 2015, with an operational profit of 6.6 billion yuan (approximately 1 billion US dollars). In 2016, the ticket sale income of the Chinese railway attained 281 billion yuan (approximately 41 billion US dollars), exceeding the freight transpiration income for the first time. Train ticket reservation has become convenient with the development of online platforms and mobile applications. Before 2011, train tickets were sold separately by railway stations and authorized train ticketing agents. The China Railway Corporation launched 12306.cn (the official ticket online reservation service), which contains a reservation management system to handle ticket booking requests in China. Online booking has become the main way to buy tickets.

Centralized ticket reservation management enables railway operators to apply demand-side management means to improve the total income. Revenue management is one of demand management techniques for maximizing the total revenue by finding an optimal strategy of control the availability and/or the price of products (e.g., train tickets) without any changes in the transportation resources (e.g., adding trains or carriages in a train). However, up to now, the train ticket prices in China are under strict supervision so that the only way for railway revenue management is to determine the availability of each product.

For most revenue management systems, the product availability is controlled via computer reservation system (CRS), because it depends on the status of all the remaining resources (i.e., how many seats are still not allocated) and need to be updated in real time. Thus, the railway revenue management can be regarded as how to set the parameters of CRS to improve the total income. Depending on the booking mechanism behind the adopted CRS, the content of decision-making (i.e., the type of parameters required by the CRS system) for different industries is usually different. Our focus is on the ticket booking mechanism of China railway (related to as the “seat-based control” mechanism), under which the parameters for the CRS is the seat allcation for each bucket (the mechanism will be introduced in section 3).

The decision-making of revenue management is based on customer demand forecast, in which an important factor is the customer number of different Origin-Destinations. With the application of CRS, the railway companies are able to collect more information about customer behavior, such as the customer arrival time (the time when a customer starts to book a ticket) [1] and their purchase preference [2]. Recently, more and more researches proved that customer behavior is also an important factor affecting the ticket sale profit [3, 4].

This paper aims to optimize the seat allocation for China railway with consideration of custom er behavior. To evaluated the ticket sale revenue with given seat allocation, a Discrete-Time Markov Chain model, which is compatible with the customers’ behavior model, is proposed to describe the ticket reservation process under the seat-based control. A genetic algorithm is proposed to deal with the complexity of ticket booking situations. The performance of proposed method is tested in two experiments with two other benchmarks. Then we apply this method in a real world case with practical data from the Nanning-Guangzhou high-speed railway line.

The remainder of the paper is organized as follows: Section 2 introduces some terminologies of revenue management and previous studies on the optimization of different revenue management systems, such as airline revenue management systems in the U.S. Section 3 illustrates the ticket booking mechanism in China. Section 4 formulates the ticket reservation process. Section 5 develops a simulation-based genetic algorithm. Section 6 tests the method on two railway networks of different sizes and shows an application to the Nanning-Guangzhou high-speed railway line. Finally, section 7 summarizes our research.

## 2 Background and previous studies

### 2.1 Railway revenue management

The seat allocation problem belongs to the research branch of revenue management, which was derived from deregulation in the U.S. airline industry in the 1970s. The literature on revenue management in the airline industry is vast [5–7]. Studies on railway revenue management problem has been paid more and more attention [8, 9].

**Fig 1:**
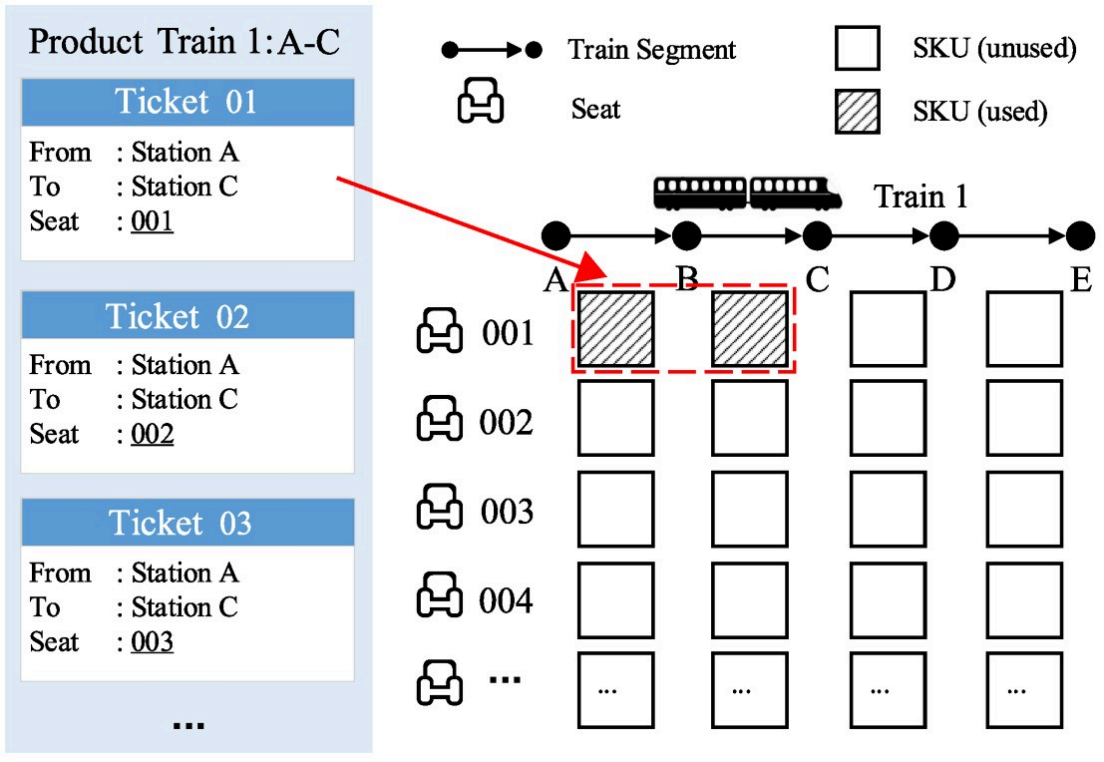
Illustration of basic elements (Reprinted from https://doi.org/10.1371/journal.pone.0201718.g001 under a CC BY license, with perimission from PLOS, orginal copyright 2018).

Some definitions relevant to railway revenue management are listed below, with examples provided in Fig 1, where a train starts from station A and stops at a sequence of intermediate stations (stations B, C, and D) before reaching terminal station E.

**Train-segment**. A train segment is the trip of a specific train between two adjacent intermediate stations.
**Stock-keeping unit (SKU)**. A stock-keeping unit is the lowest level unit of seat inventory, referring to a specific seat over a specific train segment. In Fig 1, each square represents an SKU.
**Ticket**. A train ticket contains information of the train number, the seat number, the origin station, and the destination station. It can be interpreted as the entitlement to one or more assigned SKUs. Ticket 01 in Fig 1 from station A to station C for seat No. 001 occupies the two marked SKUs.
**Product**. A product is referred to as a combination of the train number, origin station, and destination station. In the reservation process, customers will send requests for products to the reservation system. If a request is accepted, a ticket will then be printed, i.e., SKUs are assigned to sell this product. For simplicity, we use “Train 1: A-C” to indicate the product whose origin is station A and destination is station C provided by Train 1. Additionally we use “A-C” if there is only one train in the context.
**Offered product**. In the ticket reservation horizon, the railway operator will decide dynamically which products are available to customers (i.e., only the requests for those products will be accepted). The set of available products are referred to as the offered product.

The key issue of decision-making in revenue management is to evaluate the performance of a given group of input parameters of CRS. In order to do this, the most direct way is to model the ticket booking process. The ticket reservation process model should cover: 1) the ticket booking mechanism, which determines the offered products at all times in the reservation horizon; 2) the travel demand, which determines how the tickets are sold.

### 2.2 Ticket booking mechanisms

As mentioned in 1, the availability of each product is determined by a ticket booking mechanism derived from the CRS with input data such as the remaining SKUs and forecast travel demand. Based on the implementation of CRS, Talluri and Ryzin [10] classified ticket booking mechanisms into different types, such as partitioned booking limit control and Virtual Nesting. The following will briefly introduce these two mechanisms and related studies.

#### 2.2.1 Partitioned booking limit control (PBLC)

**Fig 2:**
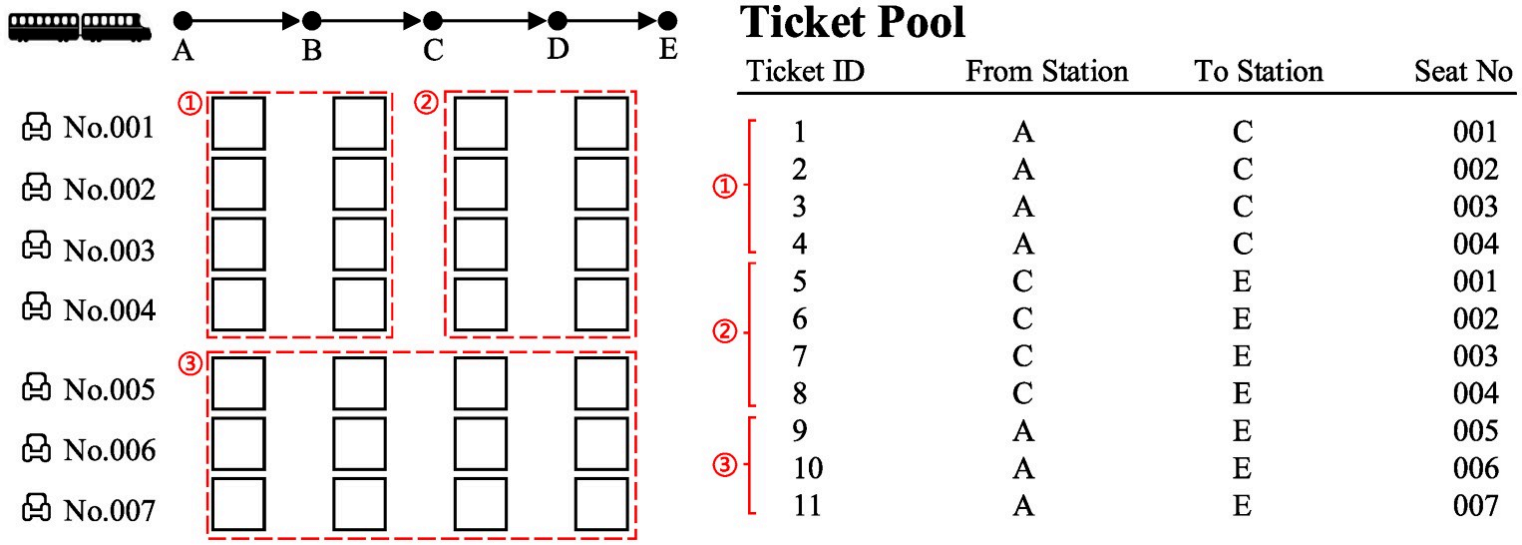
An example of partitioned booking limit control.

Partitioned booking limit control is a direct and intuitive type of ticket booking mechanism, in which the SKUs are assembled into tickets at the beginning of the booking horizon. Fig 2 shows an example of PBLC in the train of Fig 1. The SKUs from 7 seats are assembled into 3 groups, and 11 tickets are generated. PBLC has an extensive range of applications for railway companies because implementation is simple.

The main focus of studies towards PBLC is finding an optimal allocation of SKUs [8, 11–13]. However, PBLC is considered to be inefficient in practice when demand is stochastic [10]. The main reason is the fixed use of SKUs in PBLC. For example, consider that the train in Fig 1 has only one seat and only one customer exists. However, we do not know which product this costumer will request between product A-C and product A-E. Assume that the price of A-C is 50 Yuan and the price of product A-E is 100 Yuan. When adopting PBLC, two options are available: i) generating a ticket for A-E or ii) generating a ticket for A-C and a ticket for C-E. When adopting an SKU-flexible control, tickets are generated according to the order of arrival of customers. Table 1 shows the revenues of two situations. Situation 1 presents the case when the customer chooses product A-E, and situation 2 presents the case when the customer chooses product A-C. The flexible use of SKUs obviously achieves a better performance.

**Table 1:** Example of the advantage of seat-based control.

Control Pattern	Revenue in Situation 1	Revenue in Situation 2
PBLC (one ticket for A-E)	100	0
PBLC (one ticket for A-C and one ticket for C-E)	0	50
SKU-flexible control	100	50

#### 2.2.2 Virtual nesting

**Fig 3:**
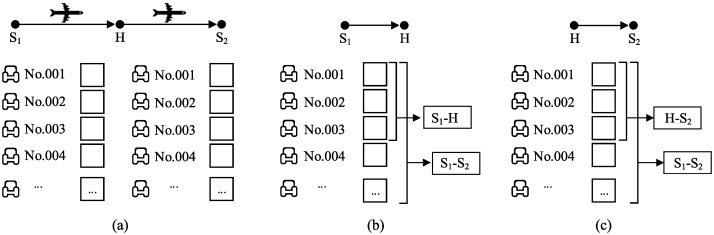
Virtual nesting control in the airline industry.

Virtual nesting is a ticket booking mechanism widely used in airline industry. It is based on the idea of allocating SKUs in a flexible manner and mainly processed by dividing the SKUs (an SKU in the airline industry is a seat over a flight segment) into independent groups by flight segment. In each group, the SKUs are assigned to products in a nested manner. A product is offered only when it is available for all of its relevant segments. Fig 3 shows an example of virtual nesting in a hub-and-spoke airline network. Assume that a joint flight is scheduled from *S*_1_ to *S*_2_ via *H*. Following the principle of virtual nesting, the SKUs in this flight are separated into 2 groups (group *S*_1_ → *H* and group *H* → *S*_2_), as shown in Fig 3(a). In the group of segment *S*_1_ → *H*, the SKUs of seats No. 001-003 are assigned to product *S*_1_−*H* and product *S*_1_ − *S*_2_, while other SKUs are assigned to product *S*_1_ − *S*_2_, as shown in Fig 3(b). A similar nested structure is set for the group of segment *H* → *S*_2_, as shown in Fig 3(c). Product *S*_1_ − *S*_2_ is offered when it is available (when unused SKUs are assigned to it) in both groups. Interested readers can refer to Talluri and Ryzin [10] for a detailed description of virtual nesting control.

The parameter setting problem for virtual nesting control is challenging. Williamson [5] claimed that simultaneously optimizing the combinations of products and the seat numbers assigned to them is both theoretically infeasible and impractical. In the research of Bertsimas and Boer [14], the combination of products is determined by a “displacement adjusted virtual nesting” (DAVN) process, which was proposed by Smith and Penn [15]. The seat number of each bucket is solved by a stochastic gradient algorithm.

### 2.3 Modeling the customer behaviors

In early studies, a group of independent, static and origin-destination-data-based demand model is usually built to make revenue management decisions. For example, Ciancimino and Inzerillo [8], You [11] proposed a randomizedlinear programming model, in which the randomness of the customer number for each origin-destination (OD) is considered. As an increasing number of the arrival time data being collected by railway enterprises, an emerging trend of studies of dynamic demand, in which the customer arrival stream is formulated as a Poisson process, has been observed. Following this idea, the revenue management was reformulated into a Markovian decision process (MDP) [16–19].

Customer choice has been recognized as an important factor that leads to correlation between the demand for each ticket [20]. Hetrakul [21] reported that a high yield is provided by the revenue management strategy, which accounts for customer choice behavior. The integration of modeling revenue management problem and customer choice has been reported in many studies. In previous studies, the assumption of independent demand was always made. Then, customer choice behavior is modeled with an introduction of the market segmentation, in which customers from each market segment may choose from a set of alternative products. Talluri and Ryzin [22] embedded a general discrete choice model into the DTMC model and obtained a special “efficient set” form of the optimal solution. Gallego et al. [23] extended the DLP model with customer choice behavior and proposed an approximate choice-based linear programming (CDLP) model. Liu and Ryzin [24] applied a multinomial logit (MNL) model with disjoint market segments and solved the approximate problem using a column generation method. Bront and Vulcano [25] demonstrated that the subproblem is NP-hard and developed a greedy heuristic method to overcome its complexity. Hosseinalifam et al. [26] proposed another heuristic method in combination with the Dinkelbach algorithm.

### 2.4 Contributions

This paper attempt to find the optimal initial parameters of seat-based control with the consideration of customer behavior, including arrival time and purchase choice. We formulate the ticket reservation process with a Discrete-Time Markov Chain (DTMC) model, which is capable of integrating the customer behavior (customer arrival model and customer choice model) and seat-based control mechanism. The substantial contributions of this study to the literature on railway seat inventory control are listed below.

In practise, we introduced and discussed the seat-based control, a type of ticket booking mechanism which is rarely investigated in previous studies, but has wide application in China as well as the potential for use in other railway operators. The seat-based control has more advantages in impoving the profit than the PBLC for railway companies due to higher SKU-utilization efficiency.

In theroy, firstly, compared to the previous studies in railway revenue management, the new model provides a way to describe complex ticket booking mechanisms, under which we can not evaluate the sales of each product by simply calculating the gap between the quantity of resource and demand [8, 13]. In contrast, our model treats ticket reservation as a time-dependent process so that the state changes of CRS can be recorded.

Secondly, the demand model, which covers passenger arrival and purchase behavior, has been proposed in the researches of real-time optimal control problem [24, 25, 27]. This paper applied the demand model into solving the railway seat allocation problem, which further expands the application range of the passenger arrival and purchase model.

At last, we design a genetic algorithm with special construction of genes to garentee the feasibility of solution to solve the seat allcation problem due to the complexity of the proposed model. This method can explore the product combinations and the seat number assignment simultaneously, with the guarantee of a feasible solution. The performance of our method is validated by comparison with two other benchmarks, namely, PBLC and first-come-first-serve control (FCFSC).

## 3 The ticket booking mechanism in China

The ticket booking mechanism in China railway, related to as “seat-based control”, enables the SKUs of a seat to be used to sell tickets with more possible combinations, which increases the probability of revenue improvement. It is designed to connect an SKU to multiple products in an alternative product set, depending on the reservation request. We refer to the book [28] (Chapter 4, in Chinese) to illustrate the mechanism for seat-based control, as it reports the implementation of CRS in China railway.

### 3.1 The joint seat regulation

**Fig 4:**
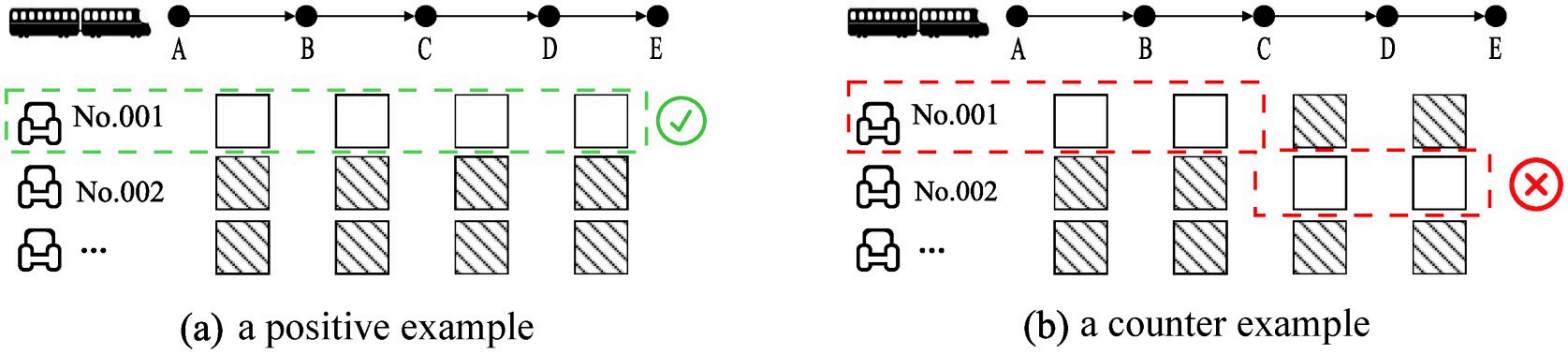
An illustration of joint seat regulation.

Before starting to introduce the seat-based control, we need to point out the joint seat regulation, a different feature of railway from the airline industry. As we all know, a train usually has several stops. So that a train ticket is related to multiple SKUs. The joint seat regulations is that the SKUs must be provided by one seat, otherwise a passenger would change his/her seat during the itinerary. Fig 4 shows an illustration of joint seat regulations.

The joint seat regulation can be guarantee by PBLC, if capacity constraints are satisfied [8]. The seat-based control is an extension of PBLC, which is able to make sure the joint seat regulation and the flexible use of SKU. In addition, China Railway Corporation allocates a seat at the same time that a ticket is sold (intercity travel), which is different from airline travel (a passenger can pick a seat when check in) and subway (no seat allocation applied). Thus, the seat-based control is also a practical approach for real-time seat allocation.

### 3.2 Bucket and ticket pool

When adopting seat-based control, each train is independently managed. For each train, seat-based control is implemented based on two fundamental elements: the bucket and the ticket pool. A bucket consists of an offered product set and a seat set and defines which products can be sold from the SKUs from the seats in the seat set. The following regulations are established for the buckets:

- A train should be related to at least one bucket. The number of buckets in a train is fixed during the reservation horizon.
- A seat should be related to only one single bucket.
- All the seats should be assigned at the beginning of the reservation horizon.
- A product can be put in at most one product set.

A ticket pool is an implementation of PBLC. In other words, the ticket pool is a collection of tickets. The ticket pool is designed to collect the tickets generated by the reusing mechanism. The following regulations for the ticket pool are established:

- A ticket pool is set to be empty at the start of the reservation horizon.
- There is only one ticket pool for each train.
- The ticket pool has a higher priority of selling tickets than the buckets.

**Fig 5:**
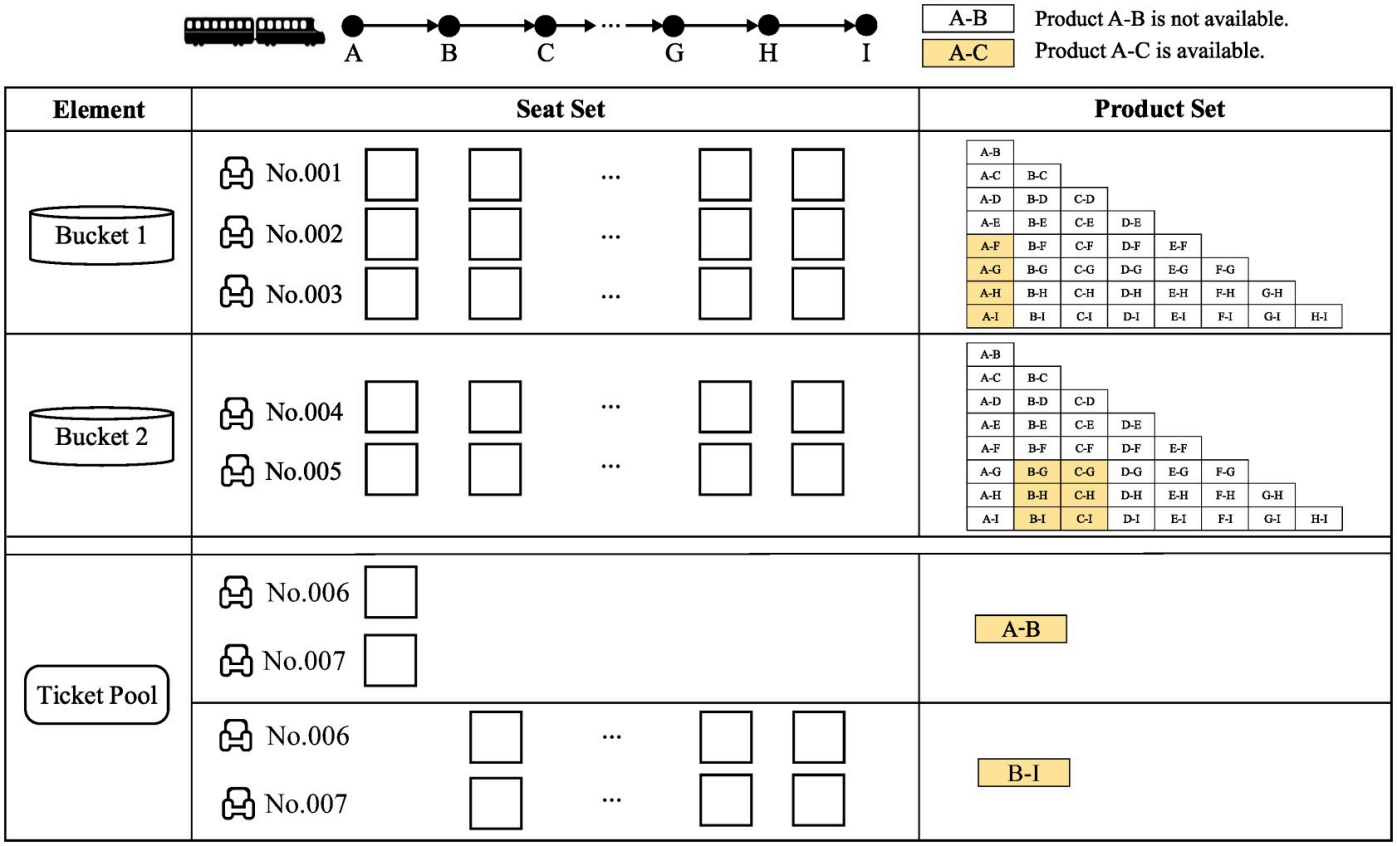
An example of the fundamental elements.

Fig 5 shows an example of a seat allocation of a train with 7 seats. There are two buckets: bucket 1 owns the SKUs of seats No. 001-003, and its offered products are products A-F, A-G, A-H and A-I; bucket 2 owns the SKUs of seats No. 004-005, and its offered products are products B-G, B-H, B-I, C-G, C-H and C-I. The ticket pool contains two tickets of both product A-B and B-I.

### 3.3 The regulations for the product set

For the customer’s ticket reservation experience, China Railway Corporation has two regulations for the product set of each bucket.

- Succession regulation. The departure/arrival stations of the offered products should be a successive sequence. For example, a product set {A-E,B-E,D-E} does not contain a successive sequence of departure stations because product C-E is absent.
- Last-station regulation. The product whose arrival station is the last station of the train should be available if any other product of the same departure station is offered. For example, if product A-B or A-C or A-D or … or A-H is available, product A-I should be available.

**Fig 6:**
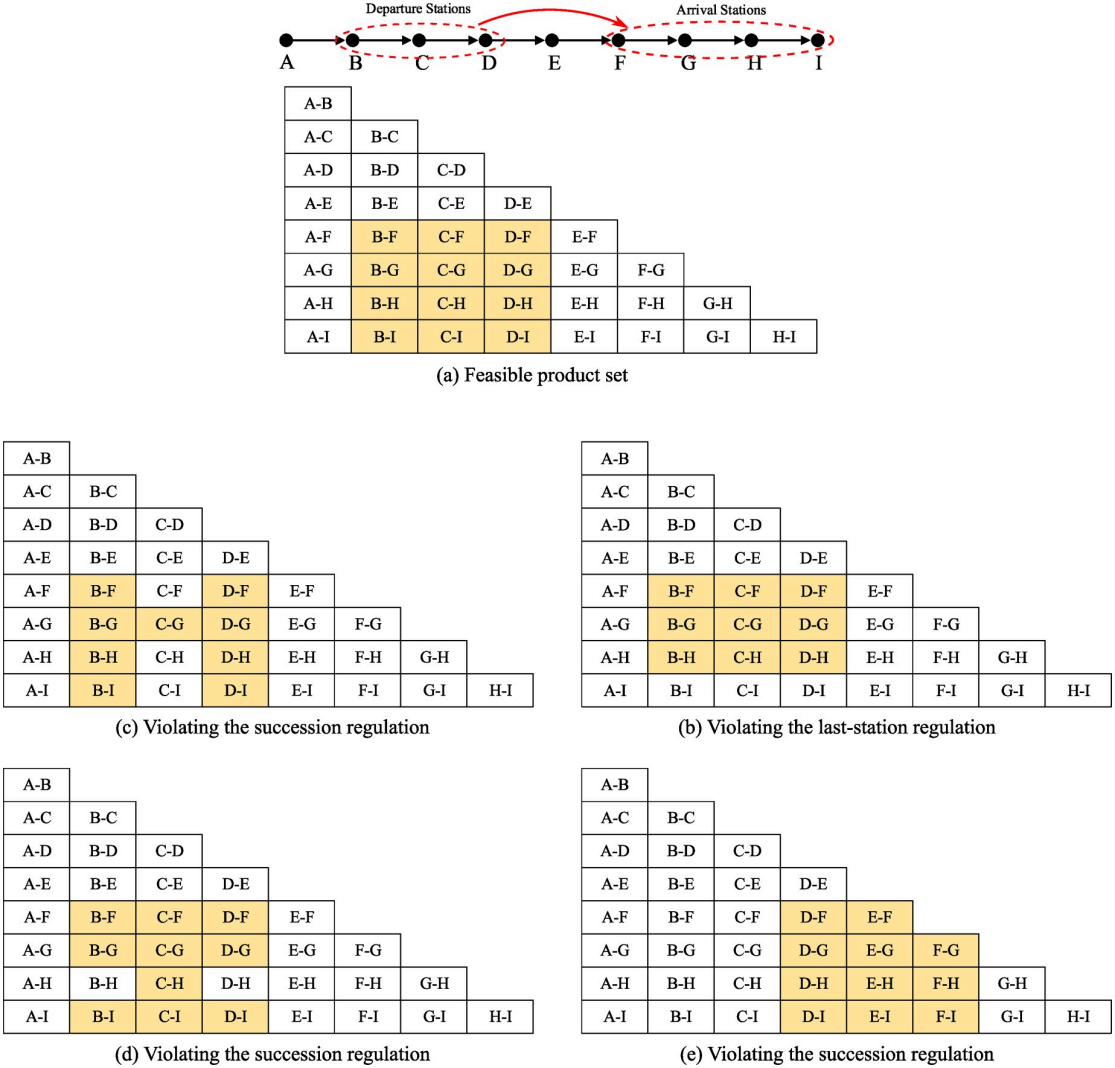
Examples of a product set.

Consider the train in Fig 5, for example. Fig 6(a) shows a feasible product set. Fig 6(b) shows an example of violating the last-station regulation. Fig 6(c) violates the succession regulation of the arrival station sequence, and 6(d) violates the succession regulation of the departure station sequence. Note that Fig 6(e) also violates the succession regulation of the station sequence, even if product F-F is not available.

### 3.4 Handling a reservation request

**Fig 7:**
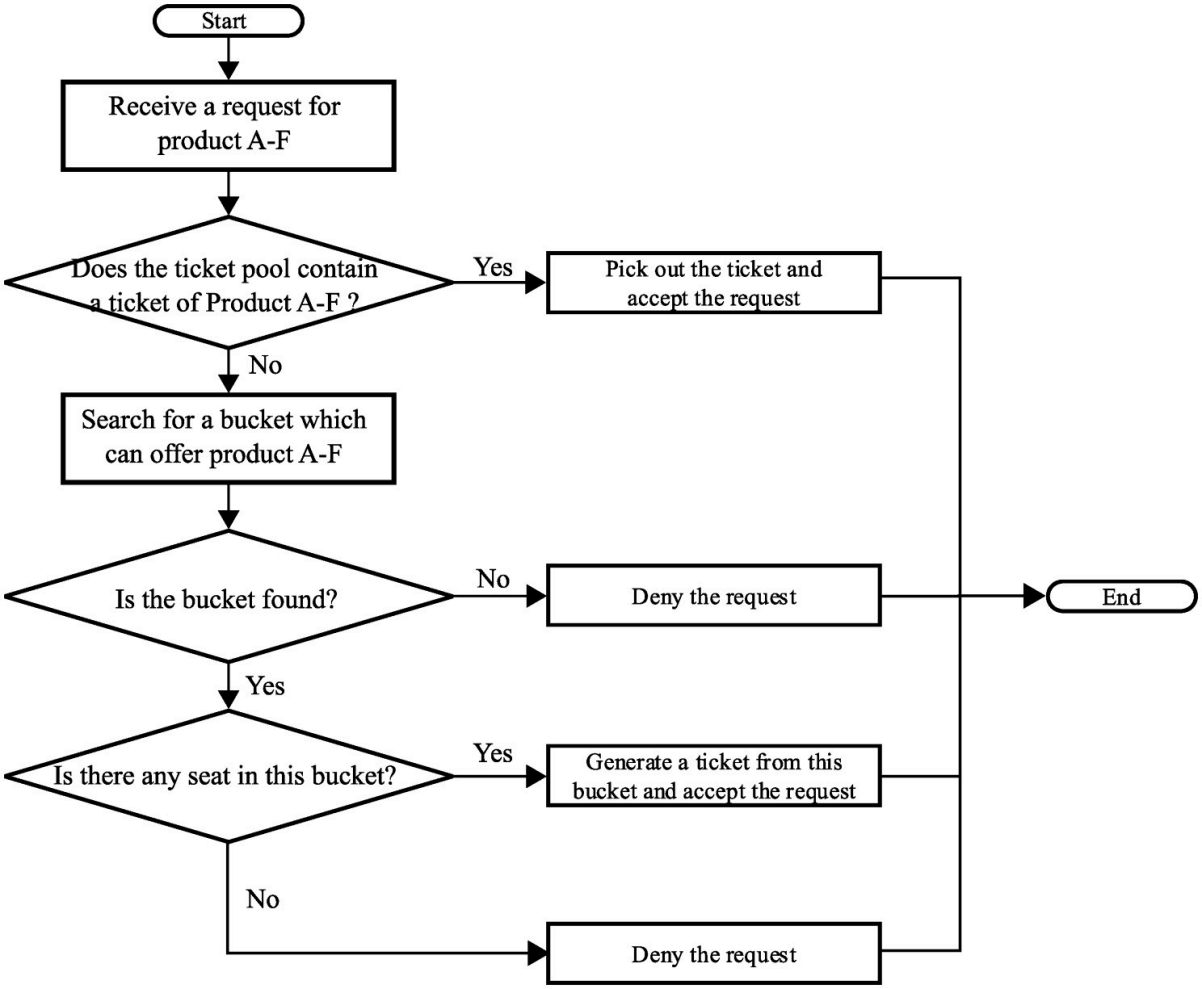
The process of handling a reservation request.

When receiving a reservation request for a product, e.g., product A-F, the reservation system handles it in the following three steps. A flow chart is also shown in Fig 7.

1. The system checks if the ticket pool contains a suitable ticket. If so, this suitable ticket will be selected. The request is then accepted with this ticket; otherwise, go to Step 2.
2. The system visits the buckets sequentially to find one bucket that can offer the requested product. If a bucket is selected, the system first chooses a seat (randomly or sequentially) and generates a ticket with the SKUs of the selected seat. The request is then accepted with the generated ticket. If no bucket is selected, proceed to Step 3.
3. The system denies the request.

### 3.5 Reusing rule of leftover SKUs

**Fig 8:**
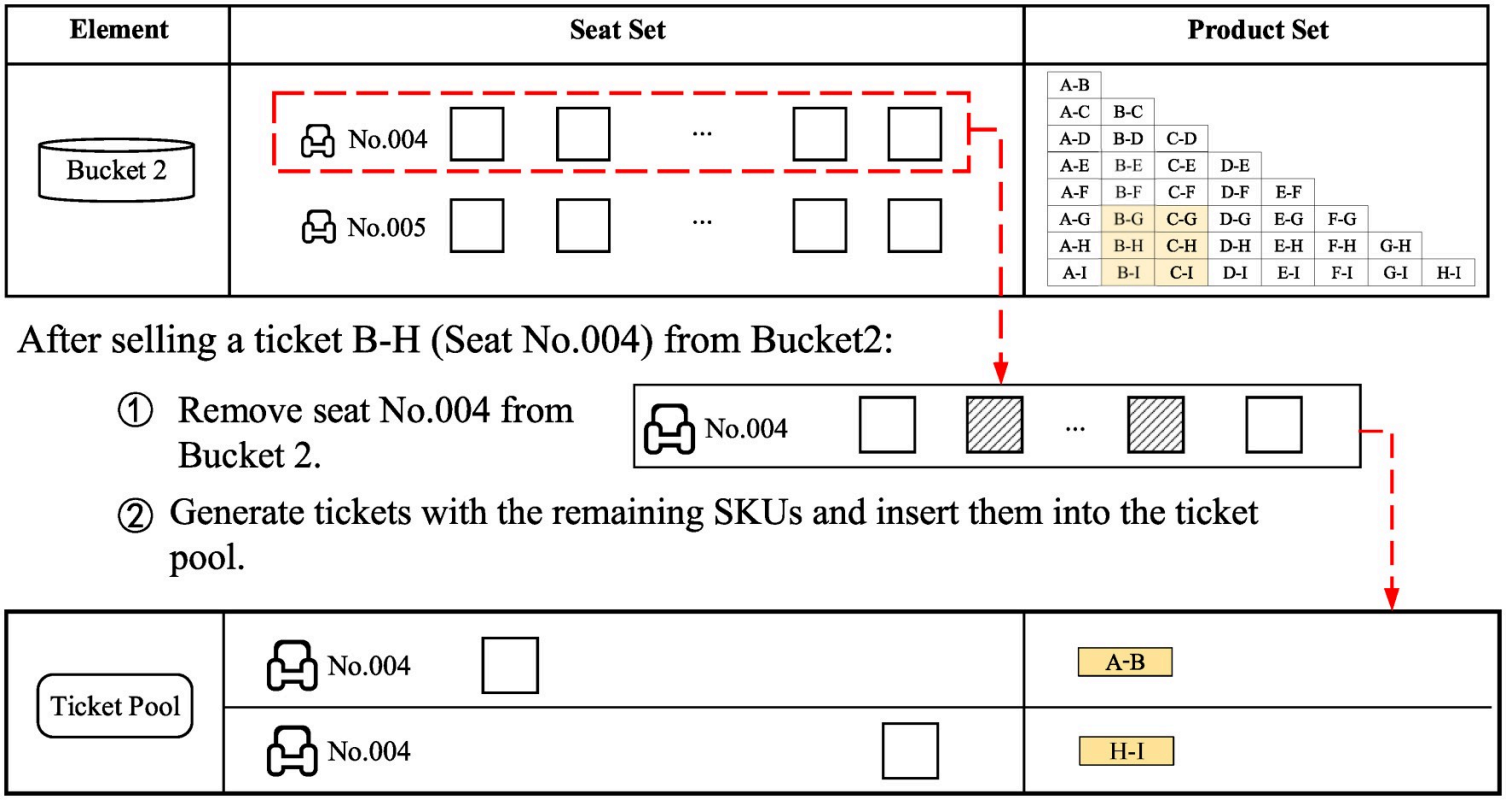
An example of reusing SKUs.

After selling a ticket from a bucket, some SKUs will remain (with the exception of the ticket for a trip from the first station to the last station). The remaining SKUs can be reused to sell additional tickets via the ticket pool. The remaining SKUs are assembled into tickets (one or two tickets depending on the pattern of the remaining SKUs). Insert the generated tickets into the ticket pool. Fig 8 shows an example of reusing the remaining SKUs of a ticket from B to H in seat No. 004. The unused SKUs will be converted to one ticket from A to B and one ticket from H to I. These two tickets will be inserted into the ticket pool. The SKU of seat No. 004 is removed from bucket 2.

## 4 Formulation

This section will formulate the seat allocation problem. For readers’ convenience, the notations are listed in Table 2.

**Table 2:** Symbol list.

Notation	Description
*T*	The time horizon, indexed by *t* = 1, …, *T*
*I*	The train set, indexed by *i*
*J*	The product set, indexed by *j*
*J*_*l*_	The consideration set of market segment *l*, where *l* ∈ *L*
*B*	The bucket set, indexed by *b*
*L*	The market segment set, indexed by *l*
*X*_*t*_	The seat number vector at epoch *t*
*Q*_*t*_	The remaining product vector at epoch *t*
***u***_*t*_	The product availability vector at epoch *t*
***x***	The initial seat number vector
***y***	The product-bucket relation vector
*δ*_*j*,*h*_	The indicator variable of the reusing relation. It equals 1 if product *j* can be sold by reusing the leftover SKUs after product *h* is sold; otherwise, it equals 0.
*r*_*j*_	The price of product *j*
*c*_*i*_	The total seat number of train *i*
$\beta_{j}^{l}$	The preference weight of product *j* for market segment *l*
*ρ*_*t*_	The probability of a customer arrival at epoch *t*
λ_*t*,*l*_	The probability that a customer who arrives at epoch *t* belongs to market segment *l*
*p*_*j*_(***u***_*t*_)	The probability that product *j* is sold out at epoch *t* with action ***u***_*t*_

### 4.1 The seat allocation problem

We make the following two assumptions in the seat allocation porblem.

- The amount of SKUs is assumed to be fixed during the reservation horizon. There is no supply adjustment of the stop stations or the number of seats in a train.
- No overbooking is considered. The offered products are limited by the remaining SKUs. In other words, a ticket cannot be sold with a lack of available SKUs.

Let *I* denote the set of trains, indexed by *i* ∈ *I*. The total seat number on train *i* is denoted by *c*_*i*_. Let *B*_*i*_ denote the set of buckets of train *i*, indexed by *b* ∈ *B*_*i*_. The entire bucket set is denoted by *B*, *B*_*i*_ ⊂ *B*, ∀ *i* ∈ *I*. Let *J* denote the set of products, indexed by *j* ∈ *J*. To start the ticket reservation, China Railway Coroperation need to determine two clusters of the parameters at the beginning of the reservation horizon for each train: (1) the number of seats of each bucket *x*_*b*_ and (2) the product set of each bucket, denoted by the the product-bucket relation *y*_*b*,*j*_ (we define *y*_*b*,*j*_ equals to 1 if product *j* is in the offered product set of bucket *b*).

The seat allocation problem is to maximize the total revenue *R*(***x***, ***y***) by finding an optimal parameter conbination (***x***, ***y***), which can be formulated into a mathematical programming form as follows:

$$\begin{array}{cc} & \underset{x,y}{\text{max}}\;R(x,y) \end{array}$$

$$\begin{array}{cc} & s.t.\\ & \sum_{b\in B_{i}}x_{b}=c_{i}\;\;\forall i\in I \end{array}$$ (1)

$$\begin{array}{cc} & \sum_{j\in J}y_{b,j}\leq 1\;\;\forall b\in B \end{array}$$ (2)

$$\begin{array}{cc} & y\in Y \end{array}$$ (3)

$$\begin{array}{cc} & x_{b}\in Z^{+}\;\;\forall b\in B \end{array}$$

$$\begin{array}{cc} & y_{b,j}=\{0,1\}\;\;\forall b\in B,\forall j\in J \end{array}$$

Constraint (1) is the capacity constraint, which ensures that every single seat is placed in one and only one bucket. Constraint (2) represents that a product can be put in at most one bucket. Constraint (3) represents the product set regulation claimed in section 3.3.

### 4.2 The ticket reservation process model

The next step of seat allocation problem is to calculate *R*(***x***, ***y***), in which the key issue is to write the analytical form *R*(***x***, ***y***). The ticket reservation process is driven by the arrival of individual customers. The sequence of customer arrival can be approximated as Bernoulli processes if an appropriate sampling time is given [29]. The Bernoulli approximation allows us to model the ticket reservation process with seat-based control as a discrete-time markov process.

Following this idea, we divide the ticket reservation horizon into *T* epochs. In epoch *t*, at most one customer will start to book ticket. The system (CRS) state at epoch *t* is denoted by a tuple (*X*_*t*_, *Q*_*t*_). *X*_*t*_ denotes the seat number vector, where *X*_*t*,*b*_ represents the number of seats in bucket *b* at epoch *t*. *Q*_*t*_ denotes the remaining product vector in the ticket pool, where *Q*_*t*,*j*_ represents the remaining number of product *j*.

The availability of products at each epoch *t* is denoted by a 0-1 vector ***u***_*t*_, where *u*_*t*,*j*_ = 1 if product *j* is offered at epoch *t*. It is a function (refered to as the “open rule”) of the state variable (*X*_*t*_, *Q*_*t*_) and the product-bucket relation vector ***y***, which is represented as (4).

$$\begin{array}{c} u_{t,j}=\{\begin{array}{cc} 1 & \sum_{b\in B}X_{t,b}y_{b,j}>0\;\text{or}\;Q_{t,j}>0\\ 0 & \text{otherwise} \end{array} \end{array}$$ (4)

- *Q*_*t*,*j*_ > 0 indicates that product *j* is available in the ticket pool.
- $\sum_{b\in B}X_{t,b}y_{b,j}>0$ means that product *j* is offered by a bucket.

With given state (*X*_*t*_, *Q*_*t*_) in current epoch *t*, there are |*J*| + 1 possible states for the next epoch. The state at epoch *t* + 1 depends on which product is sold at epoch *t*. The state transition rule can be written as (5) and (6).

$$\begin{array}{c} X_{t+1,b}=\{\begin{array}{cc} X_{t,b}-1 & \text{if}\;\text{product}\;j\;\text{is}\;\text{sold}\;\text{at}\;\text{epoch}\;t\;\text{and}\;Q_{t,j}=0\;\text{and}\;y_{j}^{b}=1\\ X_{t,b} & \text{otherwise} \end{array} \end{array}$$ (5)

$$\begin{array}{c} Q_{t+1,j}=\{\begin{array}{cc} Q_{t,j}-1 & \text{if}\;\text{product}\;j\;\text{is}\;\text{sold}\;\text{at}\;\text{epoch}\;t\;\text{and}\;Q_{t,j}>0\\ Q_{t,j}+1 & \text{if}\;\text{product}\;h\;\text{is}\;\text{sold}\;\text{at}\;\text{epoch}\;t\;\text{and}\;Q_{t,h}=0\;\text{and}\;\delta_{j,h}=1\\ Q_{t,j} & \text{otherwise} \end{array} \end{array}$$ (6)

- *δ*_*j*,*h*_ represents that product *j* can be sold by reusing the leftover SKUs when product *h* is sold. For example, in Fig 8, a ticket of product A-B and a ticket of product H-I can be sold by reusing the leftover SKUs from selling product B-H, which is denoted by *δ*_*A*−*B*,*B*−*H*_ = 1 and *δ*_*H*−*I*,*B*−*H*_ = 1.

**Fig 9:**
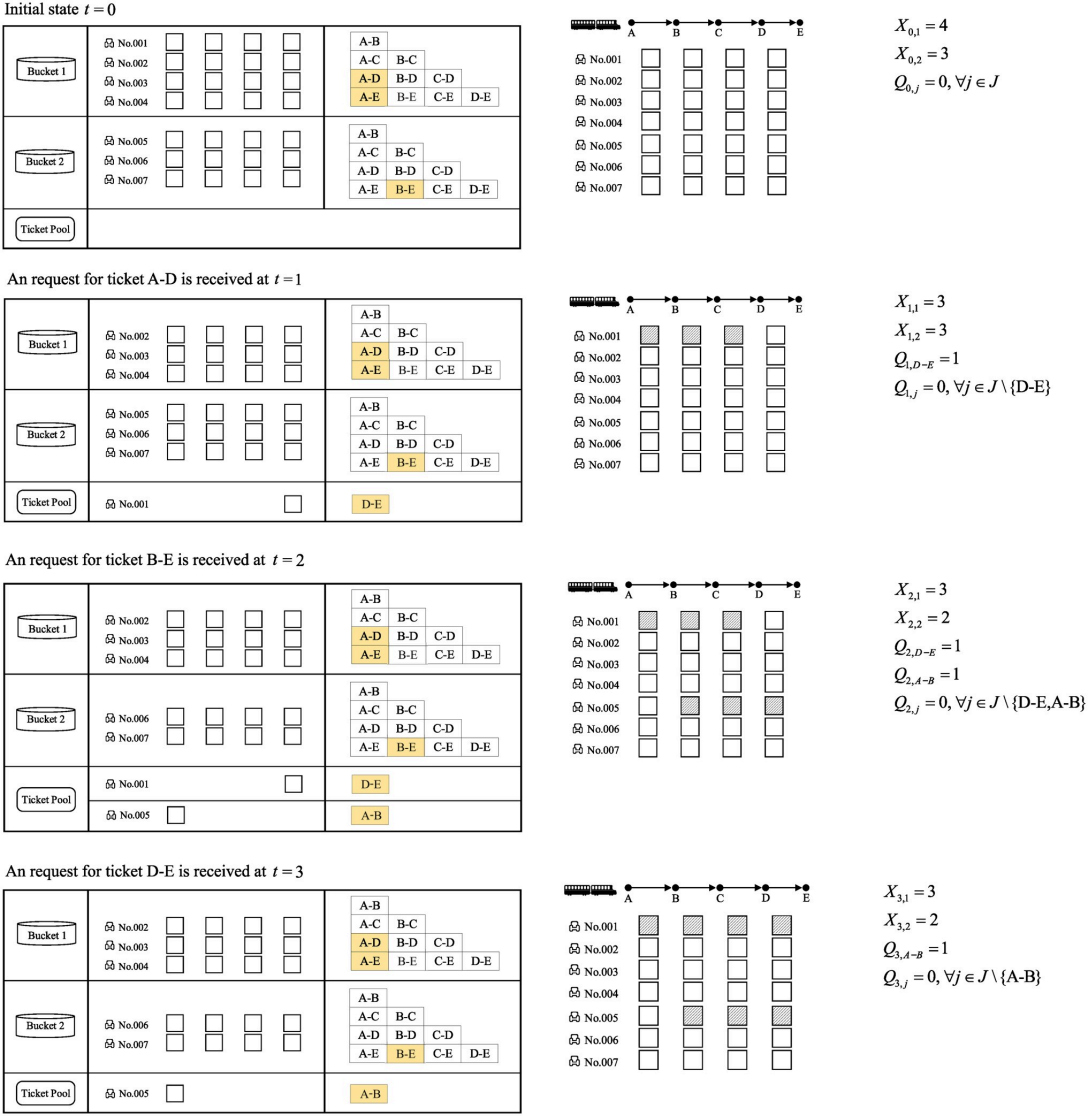
An illustration of the state transition in the DTMC model.

To illustrate the transition of state variables and the occupation of SKUs during the reservation process, we consider an example with the train in Fig 1. Assume that the reservation starts at epoch *t* = 0 and that three sequential customers arrive at *t* = 1, 2, 3 with requests for products A-D, B-E and D-E. The state variables are tracked in Fig 9.

- At epoch *t* = 0, bucket 1 has 4 seats and bucket 2 has 3 seats, which is represented by *X*_0,1_ = 4, *X*_0,1_ = 3. The ticket pool is empty; thus, *Q*_0,*j*_ = 0, ∀*j* ∈ *J*.
- At epoch *t* = 1, a ticket A-D on seat 001 is sold from bucket 1. A ticket D-E on seat 001 is generated by reuse. *X*_1,1_ = *X*_0,1_ − 1 = 3, and *Q*_1,*D*−*E*_ = *Q*_0,*D*−*E*_ + 1 = 1. Other state variables remain unchanged.
- At epoch *t* = 2, a ticket B-E on seat 006 is sold from bucket 2. A ticket A-B on seat 006 is generated by reuse. *X*_2,2_ = *X*_1,2_ − 1 = 2, and *Q*_2,*A*−*B*_ = *Q*_1,*A*−*B*_ + 1 = 1. Other state variables remain unchanged.
- At epoch *t* = 3, a ticket D-E on seat 006 is sold from the ticket pool. *Q*_3,*D*−*E*_ = *Q*_2,*D*−*E*_ − 1 = 0. Other state variables remain unchanged.

Let *r*_*j*_ denote the price of product *j*. It is fixed during the ticket reservation process. The ticket sale income at epoch *t* is denoted by *R*_*t*_ and its value can be calculated by (7).

$$\begin{array}{c} R_{t}=\{\begin{array}{cc} r_{j} & \text{if}\;\text{product}\;j\;\text{is}\;\text{sold}\;\text{at}\;\text{epoch}\;t\\ 0 & \text{if}\;\text{no}\;\text{product}\;\text{is}\;\text{sold}\;\text{at}\;\text{epoch}\;t \end{array} \end{array}$$ (7)

At the start of the booking horizon, the ticket pool is empty and the initial seat number in the bucket is ***x***; thus, *X*_0_ = ***x*** and *Q*_0_ = **0**. Thus *R*(***x***, ***y***) is equivalent to the expectation of the overall income of all epochs, which is formulated as (8).

$$\begin{array}{c} R(x,y)=E\{\sum_{t=0}^{T}R_{t}|{}_{X_{0}=x,Q_{0}=0}\} \end{array}$$ (8)

In order to calculate value of *R*(***x***, ***y***), it is necessary to calculate the probability distribution of the random variable *R*_*t*_. Further, we need to know the probability that product *j* is sold out at epoch *t* with action ***u***_*t*_, which is denoted by *p*_*j*_(***u***_*t*_). Naturally, the probability of no sale can be represented by $1-\sum_{j\in J}p_{j}(u_{t})$. The next subsection will give the analytical form of *p*_*j*_(***u***_*t*_) with the demand model.

### 4.3 The demand model

As we assumed in the last subsection, the customer arrival can be approximated as a Bernoulli processes. Let *ρ*_*t*_ denote the probability of a customer arrival at epoch *t*. So that *p*_*j*_(***u***_*t*_) can be calculated as (9), where $p_{j}^{\prime }\left(u_{t}\right)$ represents the probability of a customer selects product *j* from the available product vector ***u***_*t*_.

$$\begin{array}{c} p_{j}(u_{t})=\rho_{t}p_{j}^{\prime }\left(u_{t}\right) \end{array}$$ (9)

In marketing science, the entire market is divided into several segments to describe the heterogeneity among customers (known as “market segmentation”). We follow this idea and consider travelers’ OD combinations as the characteristic to perform segmentation. This segmentation is mutually exclusive because a customer can only belong to one OD combination, which indicates that each market segment is independent. Let *l* denote a market segment, and let *L* denote the full market. In each market segment, customers have identical behavioral patterns. Let λ_*t*,*l*_ denote the probability that a customer who arrives at epoch *t* belongs to market segment *l*. So that $p_{j}^{\prime }\left(u_{t}\right)$ can be written as (10), where *p*_*l*,*j*_ (***u***_*t*_) denotes the probability that a customer from market segment *l* selects product *j* from the available product vector ***u***_*t*_.

$$\begin{array}{c} p_{j}^{\prime }(u_{t})=\sum_{l\in L}\lambda_{t,l}p_{l,j}\left(u_{t}\right) \end{array}$$ (10)

A lot of existing discrete choice models can be applied to define *p*_*l*,*j*_(***u***_*t*_). In our research, an MNL model is adopted, which is formulated as (11). The model can be substituted with any other discrete choice models, such as the finite-mixture logit model [22] or the ordered preference list model [30].

$$\begin{array}{c} p_{l,j}(u)=\frac{u_{j}\beta_{j}^{l}}{\sum_{h\in J_{l}}u_{h}\beta_{h}^{l}+\beta_{\emptyset }^{l}},\forall l\in L \end{array}$$ (11)

- $\beta_{j}^{l}$ is the preference weight of product *j* for market segment *l*, and $\beta_{\emptyset }^{l}$ is the no-purchase preference.
- *J*_*l*_ is the consideration set of market segment *l*. It represents all the sets of possible products that a customer from market segment *l* may choose.

The parameters in the demand model, namely (***ρ***, **λ**, ***β***), can be derived from the demand forecasting results [31]. A typical form of short-term forecasting is the average customer arrival number for each market segment in a time interval [2, 12, 32].

### 4.4 Extensions

#### 4.4.1 Markov decision process (MDP) model

The decision-making in railway revenue management are usually dynamic and multi-period. It means a sequential decisions need to be made during the reservation horizon. The sequential decision problem are suible to be fomulated as Markov decision process (MDP). The MDP model has been applied in intermodal freight transportation [33] and airline industry [14]. In the practice of China railway, the seat allocation is processed only once. If we relax this constraint and allow the seat allocation to be adjusted multiple times during the reservation horizon, our problem can also be formulated in MDP.

Assume the seat allocation will be adjusted for *N* times, the reservation horizon can be divided in to *N* periods by the decision point. The Bellman’s equation is written as (12).

$$\begin{array}{c} V_{n}(X_{n},Q_{n})=\underset{x,y}{max}E\{R_{n}+V_{n+1}\left(X_{n+1},Q_{n+1}|X_{n},Q_{n},x,y\right)\} \end{array}$$ (12)

- *R*_*n*_ is a random variable, representing the income of the *n*th period. Its value depends on *X*_*n*_, *Q*_*n*_, ***x***, ***y*** and the demand.
- *V*_*n*_(*X*_*n*_, *Q*_*n*_)is the cost-to-go function, representing the sum of revenue in period *n*, *n* + 1, …, *N*.
- *X*_*n*_, *Q*_*n*_ are the state variables in the beginning of period *n*.

From the perspective of MDP, the seat allocation problem in this subsection is a one-period MDP problem. But the ticket reservation process model and the demand model still works in MDP, as they can evaluate the probability distribution of *R*_*n*_, *X*_*n*+1_ and *Q*_*n*+1_.

#### 4.4.2 Other reservation process model

Note that the ticket reservation process model can also be used to describe any other ticket booking mechanism, with redefined (1) state variables, (2) open rule and (3) state transition rule(s). For example, if we apply the DTMC model to describe the PBLC mechanism,

- the state variable is denoted by *Q*_*t*_,
- the open rule is $u_{t,j}=\{\begin{array}{cc} 1 & \text{if}Q_{t,j}>0\\ 0 & \text{otherwise} \end{array}$,
- the state transition rule is $Q_{t+1,j}=\{\begin{array}{cc} Q_{t,j}-1 & \text{if}\;\text{product}\;j\;\text{is}\;\text{sold}\;\text{at}\;\text{epoch}\;t\;\text{and}\;Q_{t,j}>0\\ Q_{t,j} & \text{otherwise} \end{array}$.

## 5 Solution approach

Some obstacles are encountered when solving the proposed seat allocation problem. It is computationally intractable due to the following three issues:

- The objective function *R*(***x***, ***y***) can be hard to calculate via the reservation process model because of the “curse of dimensionality”. There are (|*J*| + 1)^*T*^ situations to be enumerated in calculating the reservation process model, in which |*J*| and *T* are usually very large numbers in a practical problem. For example, a train of 10 stop stations can provide 45 products. Moreover, *T* depends on the number of customers, which is usually counted in tens of thousands.
- The possible number of product set combinations is too large to be enumerated by the branch-and-bound algorithm. With the constraint (3) relaxed, the number of possible combinations of *y*_*b*,*j*_ is 2^|*B*|×|*J*|^.
- The constraint (3) is hard to write in an analytical form.

Thus, this problem cannot be solved by a commercial solver (e.g., CPLEX or GUROBI). This section proposes a genetic algorithm (GA) method to solve the seat allocation problem approximately.

### 5.1 The framework of genetic algorithm

**Fig 10:**
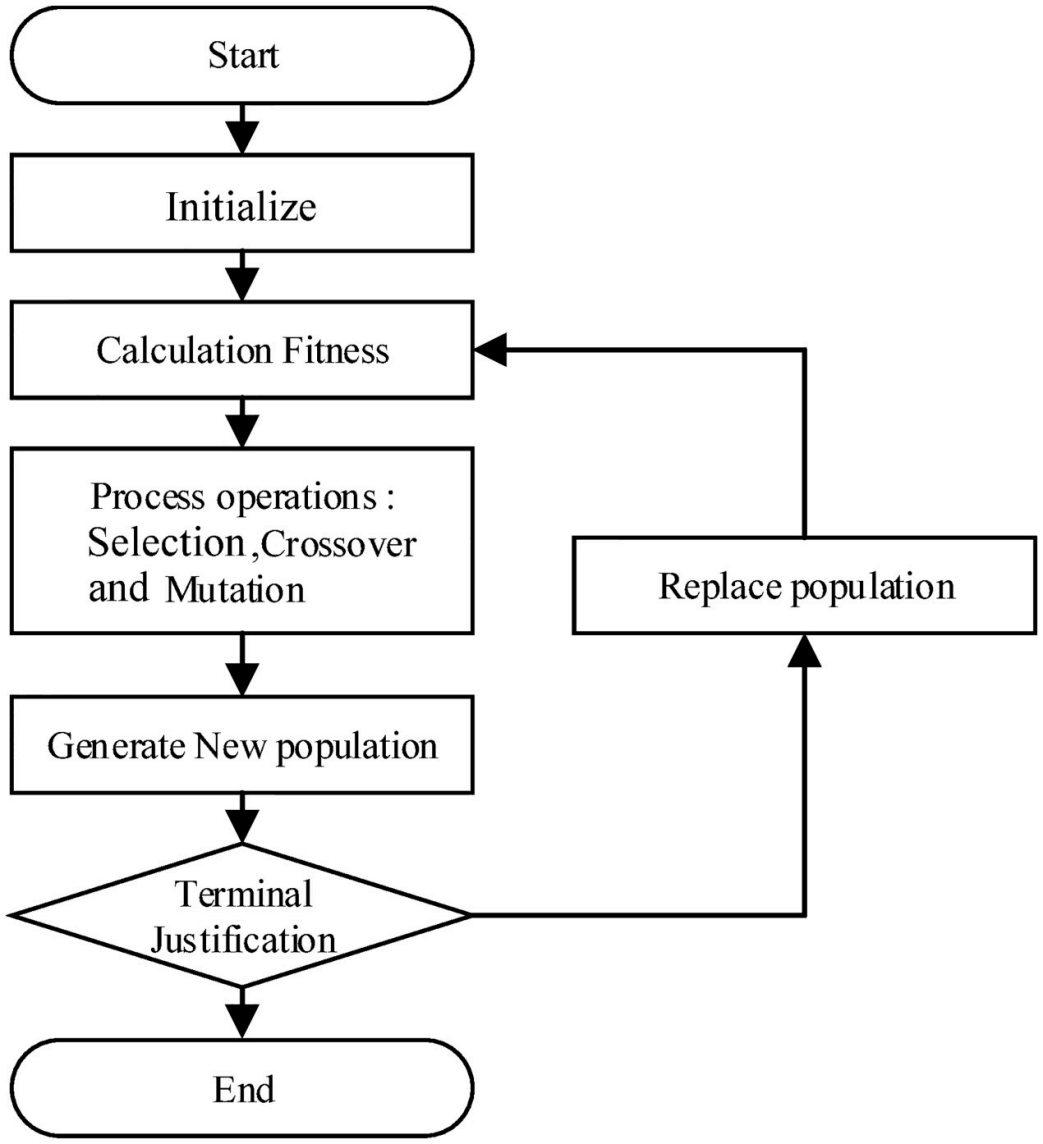
A flowchart of genetic algorithm.

GA is an adaptive heuristic search algorithm based on the evolutionary ideas of natural selection and genetics. The framework of GA is shown in Fig 10 and Alg.1. The next few subsections will elaborate each steps in details.

**Algorithm 1** The pseudo code of GA

1 Initialize:

 1.1 Generate a group of *K* chromosomes (population) encoded from random initial solution.

 1.2 Generate demand samples.

2. Calculate the fitness of each chromsome.

3. Process the following three operations: selection, crossover, mutation to generate a new group of chromosomes.

4. If the best chromosome reaches the terminal condition, then go to Step 5; otherwise replace the population and go back to Step 2.

5. Decode the current best chromosome and get a solution for the origin problem.

### 5.2 Encoding

**Fig 11:**
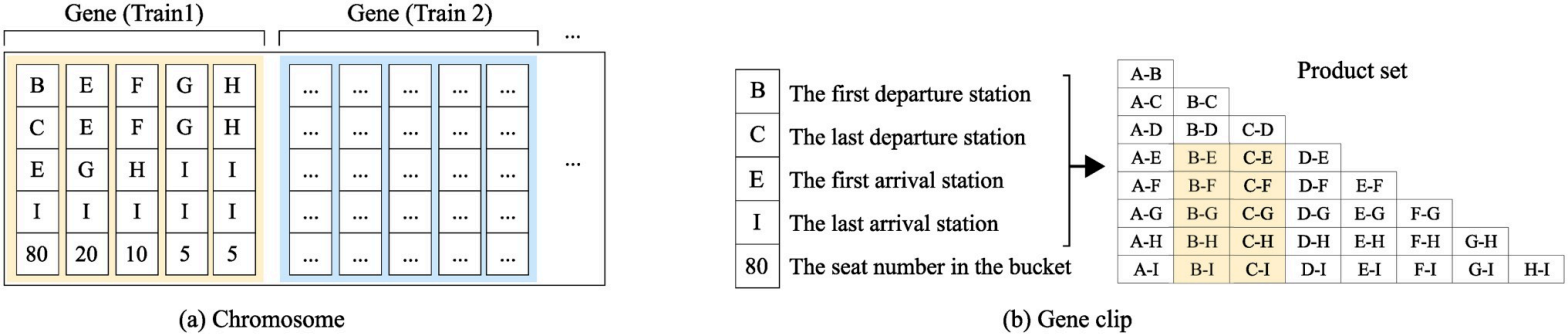
Chromosome and gene in a GA.

To adopt the genetic algorithm, a solution (*x*, *y*) is encoded to a chromosome, which consists of genes. A gene denotes the buckets of a train with some clips. Fig 11(a) shows a chromosome, in which the bucket number for each train is set to 5 (a gene has 5 clips). A gene clip denotes a bucket with 5 parameters: the first departure station, the last departure station, the first arrival station, the last arrival station and the seat number. Fig 11(b) shows a gene clip. The first 4 parameters define the product set of a bucket.

A gene clip guarantees constraint (3) by construction. For convenience, we stipulate that station A is behind station B according to the train running direction. The product set regulation is satisfied only if i) the last arrival station is set to the last station of the train and ii) the last departure station is behind the first arrival station. Constraint (1) is satisfied if the sum of the seat numbers of the gene clips is equal to the total seat number of the relevant train. Constraint (2) is satisfied if no intersection exists between the product sets defined by the gene clips.

The number of gene clips in a gene is fixed (it depends on how many buckets are supported by the reservation system). Situations in which the stop station number of a train is less than the bucket number may exist. To handle this, some virtual gene clips (empty product sets with no seats assigned) will be added.

### 5.3 Calculating fitness

The fitness is an indicator of the performance of a chromosome. In most cases, we can directly use the the objective function to evaluate the fitness. But in our problem, due to the “curse of dimensionality”, we design a statistical method to evaluate the fitness, namely the sample average appriximation (SAA) method. The basic idea of SAA is to calculate the expected total revenue by averaging income across some simulations. It has the following two steps:

The first step is to generate demand samples according to the demand parameter (***ρ***, **λ**, ***β***). A demand sample is a series of observations of random variables involved in the ticket reservation process. For the sake of comparing different solutions, we use the same demand samples in the entire solution process. The demand samples are prepared during “initialize” (Step 1.2 in Alg.1).

The second step is to simulate the ticket reservation process with the demand samples and the solution (***x***, ***y***) decoded from the current chromosome according to the ticket reservation process model and record ticket sale information. Then the fitness is calculated as the average revenue from the results.

### 5.4 Selection, crossover and mutation

We adopt the elite selection rule, which preserves the first *K* chromosomes in the population according to their fitness and deletes all subsequent ones in each iteration. So that at the beginning of each iteration, the scale of the population is fixed to *K*.

**Fig 12:**
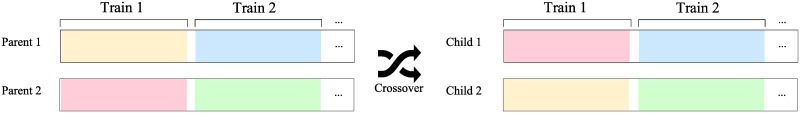
An illustration of crossover.

We adopt the uniform crossover rule, which indicates that the offspring’s gene is independently chosen from the two parents in the same location (i.e., genes representing the same train). An illustration of crossover is reported in Fig 12.

**Fig 13:**
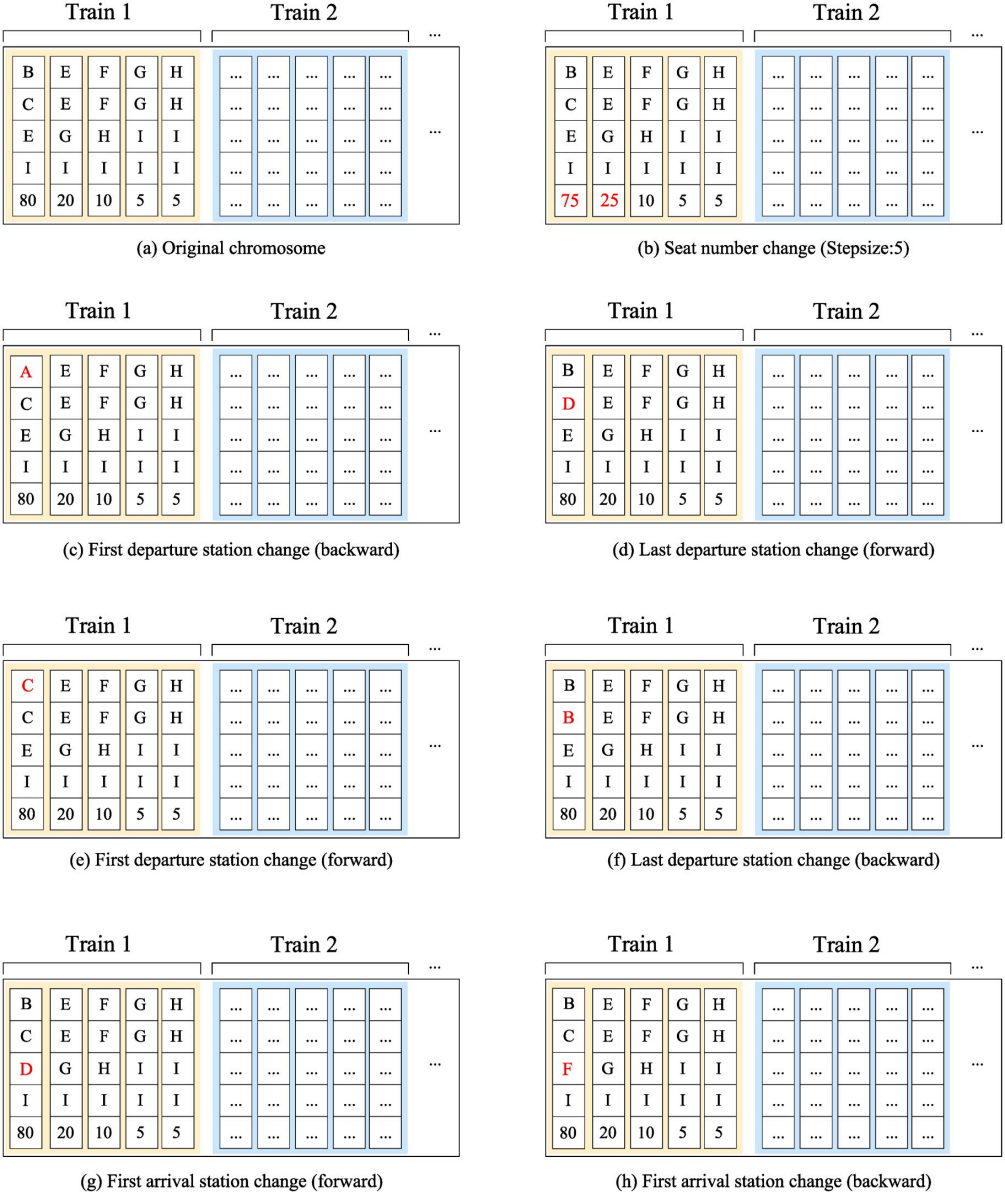
An illustration of mutation operation.

The uniform mutation rule is applied. If a gene is selected to mutate, we will randomly choose a gene clip from it and change it in one of the following mutation directions. An illustration of mutation directions are shown in Fig 13.

- Move the first departure station forward/backward.
- Move the last departure station forward/backward.
- Move the first arrival station forward/backward.
- Add/Remove *m* seat to/from the seat set (the 5th location in a gene clip), where *m* is a parameter related to specific problems.

These mutation directions can ensure that all possible solutions will be explored if there are a sufficient number of iterations. Additionally, a virtual gene clip can mutate to a normal gene clip by adding the product whose departure station is the station after the last departure station of the previous station and arrival station is the last station of the train (if this product exists).

### 5.5 Initialization

We generate the initial population of the GA by the following steps: First, for each train, we randomly pick *n* stations from the stop stations, where *n* is less than or equal to the number of buckets per train. For each selected station, a bucket is generated; the first departure station and last departure station of this product set are set to this station. Then, we randomly choose the first arrival station for each bucket. Finally, the seats are uniformly assigned to the bucket.

## 6 Numerical experiments

In this section, by using the ticket reservation process model, we evaluate different reservation scenarios through simulations. Our main concern is how the seat-based control (SBC) differs from two other ticket booking mechanism, namely the PBLC and the first-come-first-serve control (FCFSC), in the results of ticket sales. The seat allocation scheme of SBC is generated by the GA method in section 5. The maximum sale of each product in PBLC is generated by a deterministic linear programming model [8]. The first-come-first-serve control (FCFSC) sells a ticket when a sufficient number of feasible SKUs are available. In subsection 6.1, we run our experiments in two typical railway networks with different demand intensity and the number of bucket for each train. In subsection 6.2, we apply our method in a practical case of Nanning-Guangzhou high speed railway line. The algorithm and the simulation are coded in C# and run on a server (4 CPUs of 2.0 GHz, RAM 256G).

### 6.1 Test experiments

#### 6.1.1 Experiment 1: A single-train network

The network of this experiment is constructed by a train with 5 stop stations, as shown in Fig 1. The number of seats in the train is set to 40. The price of each product is fixed and listed in Table 3.

**Table 3:** Price of products.

Product	A-B	A-C	A-D	A-E	B-C	B-D	B-E	C-D	C-E	D-E
Price	100	200	300	400	100	200	300	100	200	100

We set one market segment for each OD combination. Because this case is a single-train case, an OD combination is related to only one product. The demand parameters ***ρ*** and **λ** are fixed in the time horizon; their values are listed in Table 4. Because there is only one train in this case, the value of *β* does not affect the results so that its value can be given arbitrarily.

**Table 4:** The demand parameters.

*ρ*	λ_*A*−*B*_	λ_*A*−*C*_	λ_*A*−*D*_	λ_*A*−*E*_	λ_*B*−*C*_	λ_*B*−*D*_	λ_*B*−*E*_	λ_*C*−*D*_	λ_*C*−*E*_	λ_*D*−*E*_
0.20	0.025	0.05	0.075	0.063	0.025	0.15	0.175	0.2	0.225	0.025

We process 7 cases of different demand intensities by changing the length of the time horizon *T*. The time horizon for each case is set to *T* = (100, 200, 300, 400, 500, 600, 700). The other settings are listed as follows:

- A batch of 100 demand samples is generated for each case to make sure the confidence no less than 99% (range size of confidence interval = 100).
- The maximum number of iterations is set to 100.
- The population size is set to 100.

**Fig 14:**
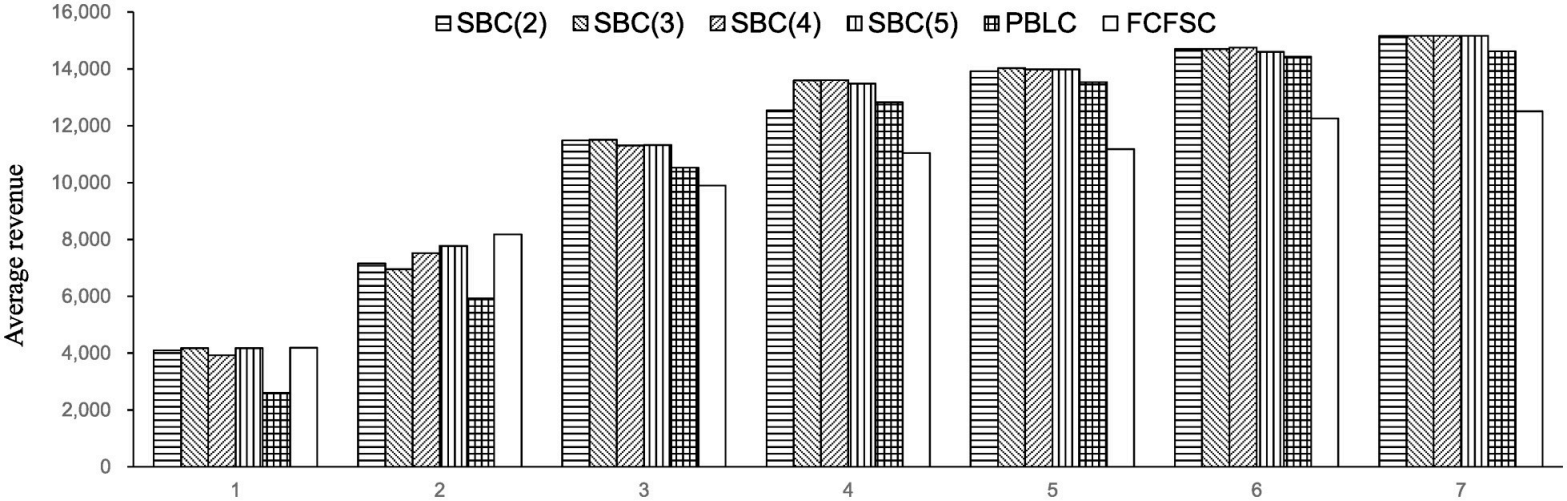
Result of experiment 1.

The average revenue results are shown in Fig 14.

- The y-axis shows the average revenue, and the x-axis shows the case number.
- “SBC(2)” represents the average revenue of seat-based control in which each train is related to 2 buckets. “SBC(3)”, “SBC(4)” and “SBC(5)” are similar.
- “PBLC” represents the average revenue of the PBLC.
- “FCFSC” represents the average revenue of the FCFSC.

#### 6.1.2 Experiment 2: A 10-train network

**Fig 15:**
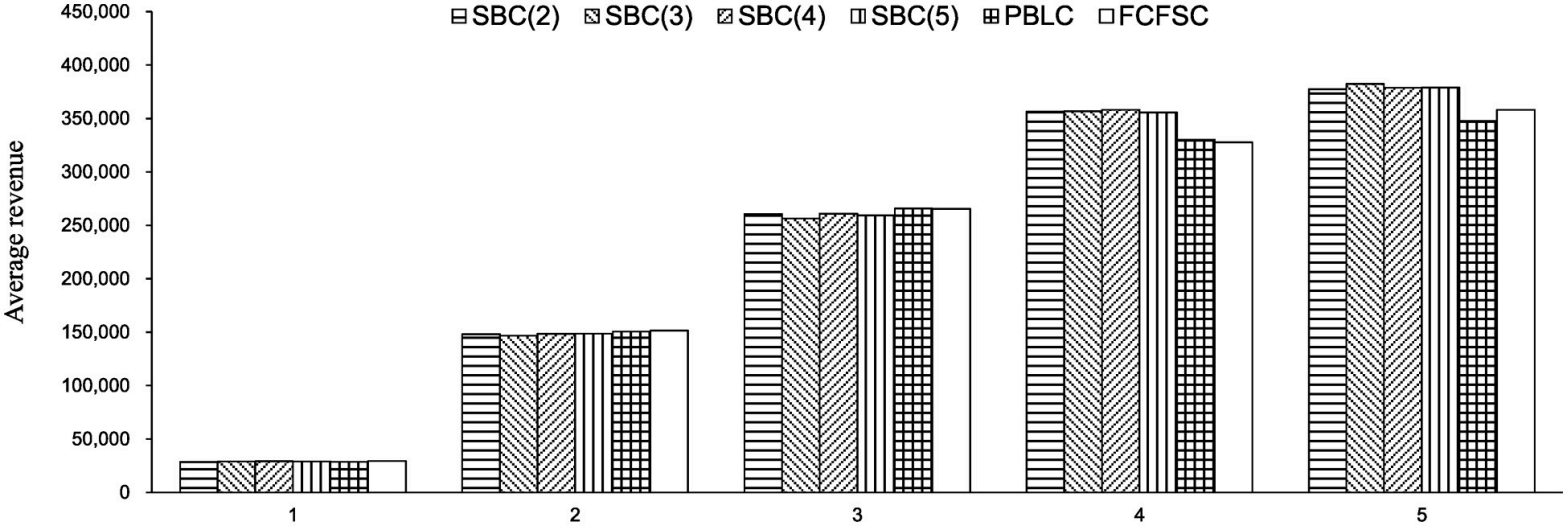
Result of experiment 2.

This experiment is based on a 10-train sub-network with 5 stations and 10 trains. Every train stops at all stations. The customer choice data are obtained from Hosseinalifam et al. [26]. (In their research, both high-price products and low-price products are considered. We only consider the high-price products.) Each train has 5 stop stations, and the customers have different preferences for each train. The demand parameters ***ρ*** and **λ** are fixed in the time horizon. We assume that passengers have no preference for the choice of each train (ignoring the impact of *β*). We set *ρ* = 0.02 and λ = 0.1 for each market segment. The seat number of each train is set to 50. We process 5 cases of different demand intensities by changing the length of the time horizon. The setting of the time horizon for each case is set to *T* = (500, 2, 500, 5, 000, 10, 000, 15, 000). Other settings are identical to the settings in experiment 1. The results are shown in Fig 15.

#### 6.1.3 Analysis of the results

To evaluate the demand intensity of each case, the load factor *γ* is introduced as a metric that reflects the matching of the demand and the resources. The calculation method of *γ* refers to Zhang and Adelman [17]. The load factor increases when the demand becomes more intense. We make two main observations from the two experiments.

- There is no distinct trend of revenue with the change of the number of buckets. Table 5 reports some fluctuations in revenue when the bucket number changes compared to SBC(5). Some large fluctuations are observed in cases 1, 2, and 4 of experiment 1. In experiment 2, the fluctuations are within 2%.
- The seat-based control brings a high revenue in comparison with PBLC and FCFSC when demand is strong. A comparison is reported in Table 6. We let SBC(5) represent the performance of seat-based control. The dominance of seat-based control becomes more remarkable when the load factor is high, which is related to cases 5, 6, and 7 in experiment 1 and cases 4 and 5 in experiment 2.

**Table 5:** The impact on revenue of changing number of buckets.

Experiment	Case	SBC(2)	SBC(3)	SBC(4)	SBC(5)
#1	1	-1.68%	0.00%	-5.88%	-
#1	2	-7.92%	-10.49%	-3.22%	-
#1	3	1.50%	1.68%	-0.04%	-
#1	4	-7.01%	0.78%	0.85%	-
#1	5	-0.46%	0.29%	0.00%	-
#1	6	0.65%	0.65%	0.99%	-
#1	7	0.00%	0.00%	0.00%	-
#2	1	-1.54%	-0.38%	0.27%	-
#2	2	-0.40%	-1.32%	-0.08%	-
#2	3	0.45%	-1.14%	0.54%	-
#2	4	0.32%	0.39%	0.73%	-
#2	5	-0.43%	0.89%	-0.07%	-

**Table 6:** Comparison of average revenue between seat-based control, PBLC and FCFSC.

Experiment	Case	*γ*	SBC(5) vs PBLC	SBC(5) vs FCFSC
#1	1	0.26	37.53%	-0.48%
#1	2	0.51	23.87%	-5.21%
#1	3	1.03	6.98%	12.51%
#1	4	1.55	4.89%	18.13%
#1	5	2.07	3.22%	20.06%
#1	6	2.58	1.20%	16.06%
#1	7	3.09	3.59%	17.54%
#2	1	0.1	1.12%	-1.12%
#2	2	0.49	-1.37%	-1.88%
#2	3	0.98	-2.46%	-2.28%
#2	4	1.96	7.15%	7.82%
#2	5	2.94	8.30%	5.51%

### 6.2 An application to the Nanning-Guangzhou high-speed railway

We apply our method to the Nanning-Guangzhou high-speed railway line, which was opened in 2014. The number of trains that run on it is increasing with the recent growth in travel demand. This experiment is based on the summer timetable of 2016, in which 49 trains and 28 ODs are involved. The reservation horizon is 60 days, which is divided into 7 intervals of unequal length. The data are reported in Supporting information. S1 File shows the average arrivals for each interval. S1 Fig shows the line plan (the stop stations and train capacity of each train). Due to the restriction of the reservation system of China Railway Corporation, at most 5 buckets can be set for one train. The bucket number for each train is set to 5.

**Fig 16:**
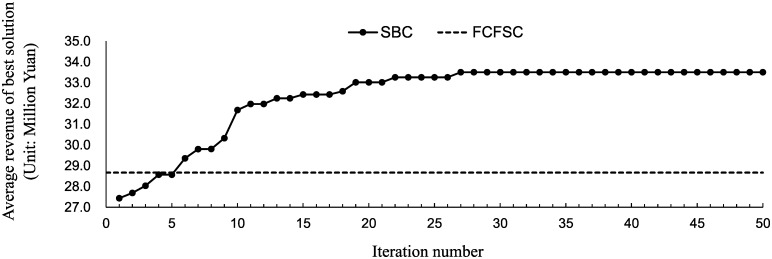
The value of the best solution in each iteration.

The solution process lasts for more than 48 hours; the best solution in each iteration is shown in Fig 16. The load factor of this case is 1.20. As indicated by the results, the average revenue of SBC exceeds that of FCFSC within 6 iterations and exceeds that of PBLC at the beginning.

Table 7 shows the final results of the two different ticket booking mechanism types. The seat-based control generates the highest revenue. We calculate three additional metrics: the average served customers (the number of customers who purchased a ticket), the average lost customers (the number of customers who did not book a ticket) and the average purchase (the average ticket fare of a sold ticket). Compared to the typical PBLC, seat-based control adds 8,000 served customers. And each customer has an extra purchase of 81 Yuan, which means there are more long-haul travelers.

**Table 7:** Results of the real world case.

Metrics	PBLC	FCFSC	SBC
Revenue (Million Yuan)	2.16	2.87	3.35
Average served customers	18,097.0	26,914.3	26,232.6
Average lost customers	19,066.3	10,249.0	10,930.7
Average purchase (Yuan)	1,196.0	1,065.1	1,277.7

## 7 Conclusion

This paper focuses on the seat allocation problem for China railway, which is a railway revenue management problem with fixed price. We specifically considered a ticket booking mechanism adopted by China Railway Corporation, called the seat-based control. In order to find the optimal combination of seat allocations, we build hierarchical models (a mathematical programming for seat allocation decision, a DTMC model for evaluating the ticket reservation process and a demand model for considering customer behavior) and then solve with a genetic algorithm. By processing simulation experiments, we compared the effects of different ticket booking mechanisms.

In terms of the implementation, the seat-based control extends the PBLC with buckets, allowing SKUs to be used flexibly. But it also makes it difficult to model the ticket reservation process. The proposed DTMC model, compared with the previous studies, is capable of revealing the complex mechanism of seat-based control. It is also compatible with the customer behavior models including the ticket reservation process model with a Bernoulli processes for the customer arrival, the market segmentation model and the choice model for purchase preference. From a practical perspective, the DTMC model enables railway companies to take full advantage of demand forecasting information.

The numerical experiments validates the performance of seat-based control by comparison with PBLC and FCFSC. The result shows that the seat-based control increased ticket sales revenue when the travel demand is strong, because it can provide more long-term travel service and reduce the unused number of SKUs.

Future research can involve the development of a faster and more efficient algorithm for this problem because a GA is time consuming in solving a real-world case; as we previously mentioned, the running time exceeds 48 hours, which may be acceptable for generating an original seat allocation. However, the algorithm cannot make timely adjustments when the demand forecast changes during the reservation process.

## Supporting information

S1 Fig BLine plan of the Nanning-Guangzhou high-speed railway. (TIF)

S1 File Dynamic demand data. (CSV)

## References

[1] Hao L, Lei N, He Z. Modeling of multi-train seat inventory control based on revenue management. In: 2016 International Conference on Logistics, Informatics and Service Sciences (LISS). 2016 International Conference on Logistics, Informatics and Service Sciences (LISS); 2016.

[2] Vulcano G, Ratliff R. Estimating Primary Demand for Substitutable Products from Sales Transaction Data. Operations Research. 2011;60(2):313–334. 10.1287/opre.1110.1012

[3] Azadeh SS, Hosseinalifam M, Savard G. The impact of customer behavior models on revenue management systems. Computational Management Science. 2014; p. 1–11.

[4] Cooper WL, de Mello TH, Kleywegt AJ. Models of the Spiral-Down Effect in Revenue Management. Operations Research. 2006;54(5):968–987. 10.1287/opre.1060.0304

[5] Williamson EL. Airline network seat inventory control: methodologies and revenue impacts; 1992.

[6] Ryzin GJV, Mcgill JI. Revenue Management: Research Overview and Prospects. Transportation Science. 1999;33(2):233–256. 10.1287/trsc.33.2.233

[7] Bertsimas D, Popescu I. Revenue Management in a Dynamic Network Environment. Transportation Science. 2003 10.1287/trsc.37.3.257.16047

[8] Ciancimino A, Inzerillo G, Lucidi S, Palagi L. A Mathematical Programming Approach for the Solution of the Railway Yield Management Problem. 1999.

[9] Armstrong A, Meissner J. Railway Revenue Management: Overview and Models. Joern Meissner. 2010.

[10] Talluri KT, Ryzin GJV. The Theory and Practice of Revenue Management. Springer Netherlands; 2004.

[11] You PS. An efficient computational approach for railway booking problems. European Journal of Operational Research. 2008;185(2):811–824. 10.1016/j.ejor.2006.12.049

[12] Jiang X, Chen X, Zhang L, Zhang R. Dynamic Demand Forecasting and Ticket Assignment for High-Speed Rail Revenue Management in China. Transportation Research Record Journal of the Transportation Research Board. 2015;2475(2475):37–45. 10.3141/2475-05

[13] Wang X, Wang H, Zhang X. Stochastic seat allocation models for passenger rail transportation under customer choice. Transportation Research Part E: Logistics and Transportation Review. 2016;96:95–112. 10.1016/j.tre.2016.10.003

[14] Bertsimas D, Boer SD. Simulation-Based Booking Limits for Airline Revenue Management. Operations Research. 2005;53(1):90–106. 10.1287/opre.1040.0164

[15] Smith BC, Penn CW. ANALYSIS OF ALTERNATE ORIGIN-DESTINATION CONTROL STRATEGIES. In: AGIFORS PROCEEDINGS; 1988.

[16] Adelman D. Dynamic Bid Prices in Revenue Management. Operations Research. 2007;55(4):647–661. 10.1287/opre.1060.0368

[17] Zhang D, Adelman D. An Approximate Dynamic Programming Approach to Network Revenue Management with Customer Choice. Transportation Science. 2009;43(3):381–394. 10.1287/trsc.1090.0262

[18] Meissner J, Strauss A. Network Revenue Management with Inventory-Sensitive Bid Prices and Customer Choice. European Journal of Operational Research. 2011;216(8):459–468.

[19] Huang K, Liang YT. A dynamic programming algorithm based on expected revenue approximation for the network revenue management problem. Transportation Research Part E: Logistics and Transportation Review. 2011;47(3):333–341. 10.1016/j.tre.2010.11.005

[20] Riss M, Cote J, Savard G. A new revenue optimization tool for high-speed railway: finding the right equilibrium between revenue growth and commercial objectives. In: Proceedings of the 8th World Congress on Railway Research, Seoul; 2008.

[21] Hetrakul P. Discrete choice models for revenue management; 2012.

[22] Talluri K, Ryzin GV. Revenue Management Under a General Discrete Choice Model of Consumer Behavior. Management Science Journal of the Institute for Operations Research & the Management Sciences. 2004;50(1):15–33.

[23] Gallego G, Iyengar G, Phillips R, Dubey A. Managing Flexible Products on a Network. 2004.

[24] Liu Q, Van Ryzin G. On the Choice-Based Linear Programming Model for Network Revenue Management. Manufacturing & Service Operations Management. 2008;10(2):288–310. 10.1287/msom.1070.0169

[25] Bront JJM, Vulcano G. A Column Generation algorithm for Choice-Based Network Revenue Management. Operations Research. 2009;57(3):769–784. 10.1287/opre.1080.0567

[26] Hosseinalifam M, Marcotte P, Savard G. A new bid price approach to dynamic resource allocation in network revenue management. European Journal of Operational Research. 2016;255(1):142–150. 10.1016/j.ejor.2016.04.057

[27] Kunnumkal S, Talluri K. Choice Network Revenue Management Based on New Tractable Approximations. Transportation Science. 2019 10.1287/trsc.2018.0867

[28] China Railway Corporation. Ticket Selling Management and Key Technology for High Speed Rails. China Railway Publishing House; 2014.

[29] Bertsekas D, Tsitsiklis JN. Introduction To Probability. Athena Scientific; 2008.

[30] Hosseinalifam M, Marcotte P, Savard G. Network capacity control under a nonparametric demand choice model. Operations Research Letters. 2015;43(5):461–466. 10.1016/j.orl.2015.06.012

[31] Lee TC, Hersh M. A Model for Dynamic Airline Seat Inventory Control with Multiple Seat Bookings. Transportation Science. 1993;27(3):252–265. 10.1287/trsc.27.3.252

[32] Bao Y, Liu J, Ma Ms, Meng Ly. Seat inventory control methods for Chinese passenger railways. Journal of Central South University. 2014;21(4):1672–1682. 10.1007/s11771-014-2109-y

[33] Wang H, Wang X, Zhang X. Dynamic resource allocation for intermodal freight transportation with network effects: Approximations and algorithms. Transportation Research Part B: Methodological. 2017;99(Supplement C):83–112. 10.1016/j.trb.2017.01.007

