# Supplementary figures and images for: Optimization of seat allocation with fixed prices: An application of railway revenue management in China
Source: PLoS One. 2020 Apr 21;15(4):e0231706. doi: 10.1371/journal.pone.0231706 (PMC7173790; doi:10.1371/journal.pone.0231706)

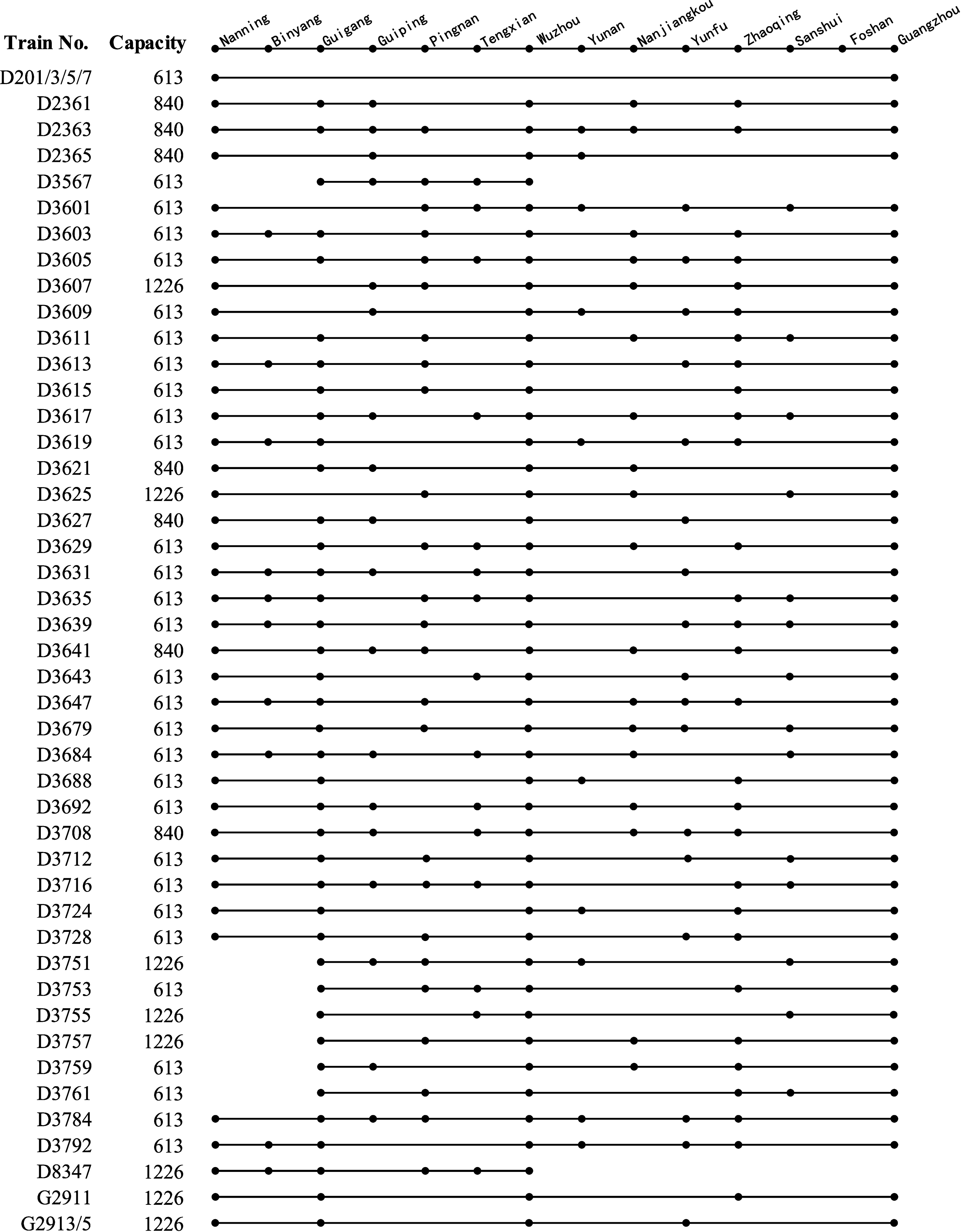

Supplement: S1 Fig — (TIF) [file pone.0231706.s001.tif]
